# The risk of COVID-19 death is much greater and age-dependent with type I IFN autoantibodies

**DOI:** 10.21203/rs.3.rs-1225906/v1

**Published:** 2022-01-14

**Authors:** Jeremy Manry, Paul Bastard, Adrian Gervais, Tom Le Voyer, Jérémie Rosain, Quentin Philippot, Eleftherios Michailidis, Hans-Heinrich Hoffmann, Shohei Eto, Marina Garcia-Prat, Lucy Bizien, Alba Parra-Martínez, Rui Yang, Liis Haljasmägi, Mélanie Migaud, Karita Särekannu, Julia Maslovskaja, Nicolas de Prost, Yacine Tandjaoui-Lambiotte, Charles-Edouard Luyt, Blanca Amador-Borrero, Alexandre Gaudet, Julien Poissy, Pascal Morel, Pascale Richard, Fabrice Cognasse, Jesus Troya, Sophie Trouillet-Assant, Alexandre Belot, Kahina Saker, Pierre Garçon, Jacques G. Rivière, Jean-Christophe Lagier, Stéphanie Gentile, Lindsey Rosen, Elana Shaw, Tomohiro Morio, Junko Tanaka, David Dalmau, Pierre-Louis Tharaux, Damien Sene, Alain Stepanian, Bruno Mégarbane, Vasiliki Triantafyllia, Arnaud Fekkar, James Heath, Jose Franco, Juan-Manuel Anaya, Jordi Solé-Violán, Luisa Imberti, Andrea Biondi, Paolo Bonfanti, Riccardo Castagnoli, Ottavia Delmonte, Yu Zhang, Andrew Snow, Steve Holland, Catherine Biggs, Marcela Moncada-Vélez, Andrés Arias, Lazaro Lorenzo, Soraya Boucherit, Dany Anglicheau, Anna Planas, Filomeen Haerynck, Sotirija Duvlis, Robert Nussbaum, Tayfun Ozcelik, Sevgi Keles, Aziz Bousfiha, Jalila El Bakkouri, Carolina Ramirez-Santana, Stéphane Paul, Qiang Pan-Hammarstrom, Lennart Hammarstrom, Annabelle Dupont, Alina Kurolap, Christine Metz, Alessandro Aiuti, Giorgio Casari, Vito Lampasona, Fabio Ciceri, Lucila Barreiros, Elena Dominguez-Garrido, Mateus Vidigal, Mayana Zatz, Diederik van de Beek, Sabina Sahanic, Ivan Tancevski, Yurii Stepanovskyy, Oksana Boyarchuk, Yoko Nukui, Miyuki Tsumura, Loreto Vidaur, Stuart Tangye, Sonia Burrel, Darragh Duffy, Lluis Quintana-Murci, Adam Klocperk, Nelli Kann, Anna Shcherbina, Yu-Lung Lau, Daniel Leung, Matthieu Coulongeat, Julien Marlet, Rutger Koning, Luis Reyes, Angélique Chauvineau-Grenier, Fabienne Venet, guillaume monneret, Michel Nussenzweig, Romain Arrestier, Idris Boudhabhay, Hagit Baris-Feldman, David Hagin, Joost Wauters, Isabelle Meyts, Adam Dyer, Sean Kennelly, Nollaig Bourke, Rabih Halwani, Fatemeh Sharif-Askari, Karim Dorgham, Jérôme Sallette, Souad Mehlal-Sedkaoui, Suzan AlKhater, Raúl Rigo-Bonnin, Francisco Morandeira, Lucie Roussel, Donald Vinh, Christian Erikstrup, Antonio Condino-Neto, Carolina Prando, Anastasiia Bondarenko, András Spaan, Laurent Gilardin, Jacques Fellay, Stanislas Lyonnet, Kaya Bilguvar, Richard Lifton, Shrikant Mane, Mark Anderson, Bertrand Boisson, Vivien Béziat, Shen-Ying Zhang, Evangelos Andreakos, Olivier Hermine, Aurora Pujol, Pärt Peterson, Trine Hyrup Mogensen, Lee Rowen, James Mond, Stéphanie Debette, Xavier deLamballerie, Charles Burdet, Lila Bouadma, Marie Zins, Pere Soler-Palacin, Roger Colobran, Guy Gorochov, Xavier Solanich, Sophie Susen, Javier Martinez-Picado, Didier Raoult, Marc Vasse, Peter Gregersen, Carlos Rodríguez-Gallego, Lorenzo Piemonti, Luigi Notarangelo, Helen Su, Kai Kisand, Satoshi Okada, Anne Puel, Emmanuelle Jouanguy, Charles Rice, Pierre Tiberghien, Qian Zhang, Jean-Laurent Casanova, Laurent Abel, Aurélie Cobat

**Affiliations:** INSERM U1163; Laboratory of Human Genetics of Infectious Diseases, Necker Branch, INSERM U1163; INSERM U1163; INSERM U1163; Laboratory of Human Genetics of Infectious Diseases, Necker Branch, INSERM; INSERM U1163; The Rockefeller University, Laboratory of Virology and Infectious Disease; Laboratory of Virology and Infectious Disease, Rockefeller University; Department of Pediatrics, Graduate School of Biomedical and Health Sciences, Hiroshima University; Pediatric Infectious Diseases and Immunodeficiencies Unit, Hospital Universitari Vall d’Hebron, Vall d’Hebron Research Institute; INSERM U1163; Pediatric Infectious Diseases and Immunodeficiencies Unit, Hospital Universitari Vall d’Hebron, Vall d’Hebron Research Institute; St. Giles Laboratory of Human Genetics of Infectious Diseases, Rockefeller Branch, Rockefeller University; University of Tartu; INSERM U1163; Institute of Biomedicine and Translational Medicine, University of Tartu; Institute of Biomedicine and Translational Medicine, University of Tartu; Hôpitaux Universitaires Henri Mondor; Avicenne Hospital, AP-HP, Bobigny, INSERM U1272, Hypoxia and Lung; Hôpital Pitié-Salpêtrière, Service de Médecine Intensive Réanimation, Institut de Cardiologie; Internal Medicine Department, Lariboisière Hospital AP-HP, Paris University; University of Lille, U1019-UMR9017, Center for Infection and Immunity of Lille; University of Lille, U1019-UMR9017, Center for Infection and Immunity of Lille; Etablissement Français Du Sang; Etablissement Français Du Sang; Etablissement Français du Sang, Auvergne-Rhône-Alpes; Department of Internal Medicine, Infanta Leonor University Hospital; Hospices Civils de Lyon; Hospices Civils de Lyon; Hospices Civils de Lyon; Intensive Care Unit, Grand Hôpital de l’Est Francilien Site de Marne-La-Vallée; Hospital Universitari Vall d’Hebron; Méditerranée Infection Foundation; Service d’Evaluation Médicale, Hôpitaux Universitaires de Marseille APHM; National Institutes of Health; Laboratory of Clinical Immunology and Microbiology, National Institute of Allergy and Infectious Diseases, National Institutes of Health,; Tokyo Medical and Dental University; Department of Epidemiology, Infectious Disease Control and Prevention, Graduate School of Biomedical and Health Sciences, Hiroshima Universit; Hospital Universitari MútuaTerrassa; Fundació Docència i Recerca MutuaTerrassa, Terrasa; Universitat de Barcelona; Institut National de la Santé et de la Recherche Médicale (INSERM); Internal Medicine Department, Lariboisière Hospital AP-HP, Paris University; Service d’Hématologie Biologique, Hôpital Lariboisière, AP-HP and EA3518, Institut Universitaire d’Hématologie-Hôpital Saint Louis, Université Paris; Réanimation Médicale et Toxicologique, Hôpital Lariboisière (AP-HP), Université Paris-Diderot, INSERM Unité Mixte de Recherche Scientifique (UMRS) 1144; Laboratory of Immunobiology, Center for Clinical, Experimental Surgery, and Translational Research, Biomedical Research Foundation of the Academy of Athens; INSERM U1163; Institute for Systems Biology; University of Antioquia; Universidad del Rosario; Intensive Care Medicine, University Hospital of Gran Canaria Dr. Negrín, Canarian Health System; CREA Laboratory (AIL Center for Hemato-Oncologic Research), Diagnostic Department, ASST Spedali Civili di Brescia; Fondazione Tettamenti; Department of Infectious Diseases, San Gerardo Hospital, University of Milano Bicocca; Laboratory of Clinical Immunology and Microbiology, National Institute of Allergy and Infectious Diseases, National Institutes of Health; Immune Deficiency Genetics Section, Laboratory of Host Defenses, Division of Intramural Research, National Institute of Allergy and Infectious Diseases, National Institutes of Health; NIAID; Uniformed Services University of the Health Sciences, Bethesda, MD; Division of Intramural Research (HNM2), National Institute of Allergy and Infectious Diseases; Department of Pediatrics, British Columbia Children’s Hospital, University of British Columbia; St. Giles Laboratory of Human Genetics of Infectious Diseases, Rockefeller Branch, Rockefeller University; St. Giles Laboratory of Human Genetics of Infectious Diseases, Rockefeller Branch, Rockefeller University; Necker Medical School; Necker Medical School; CHU Necker; Spanish National Research Council; Ghent University Hospital; Faculty of Medical Sciences, University “Goce Delchev,” Štip, Republic of Northern Macedonia.; Invitae (United States); Bilkent University; Necmettin Erbakan University, Meram Medical Faculty; Centre Hospitalier Universitaire Hassan II; Clinical Immunology Unit, Department of Pediatric Infectious Disease, CHU Ibn Rushd and LICIA, Laboratoire d’Immunologie Clinique, Inflammation et Allergie, Faculty of Medicine and Pharmacy,; Center for Autoimmune Disease Research, School of Medicine and Health Sciences, Universidad del Rosario, Bogotá, Colombia.; Centre International de Recherche en Infectiologie Lyon; Karolinska Institute; Karolinska Institute; Université de Lille, INSERM, CHU de Lille, Institut Pasteur de Lille, U1011-EGID, F-59000 Lille, France.; Genetics Institute, Tel Aviv Sourasky Medical Center, Tel Aviv University, Tel Aviv, Israel.; Feinstein Institute for Medical Research; San Raffaele Telethon Institute for Gene Therapy (SR-Tiget), IRCCS San Raffaele Scientific Institute, Milan; Vita-Salute San Raffaele University, and Clinical Genomics, IRCCS Ospedale San Raffaele, Milan, Italy.; IRCCS Ospedale San Raffaele; Hematology and Bone Marrow Transplantation Unit, IRCCS San Raffaele Scientific Institute, Milano, Italy.; Department of Immunology, Institute of Biomedical Sciences, University of São Paulo, São Paulo, Brazil.; Fundación Rioja Salud, Centro de Investigación Biomédica de La Rioja, Logroño, Spain.; University of São Paulo, São Paulo, Brazil.; University of São Paulo, São Paulo, Brazil.; Department of Neurology, Amsterdam Neuroscience, Amsterdam, Netherlands.; Department of Internal Medicine II, Medical University Innsbruck; Innsbruck Medical University; Shupyk National Healthcare University of Ukraine, Kyiv, Ukraine.; Department of Children’s Diseases and Pediatric Surgery, I. Horbachevsky Ternopil National Medical University, Ternopil, Ukraine.; Department of Infection Control and Prevention, Medical Hospital, TMDU, Tokyo, Japan.; Hirosima University; Intensive Care Medicine, Donostia University Hospital, Biodonostia Institute of Donostia, San Sebastián, Spain.; Garvan Institute; Sorbonne University; Translational Immunology Lab, Institut Pasteur; Institut Pasteur; Department of Immunology, Second Faculty of Medicine, Charles University and University Hospital Motol, 15006 Prague; Dmitry Rogachev National Medical Research Center of Pediatric Hematology, Oncology and Immunology, Moscow, Russia.; Dmitry Rogachev National Medical Research Center of Pediatric Hematology, Oncology and Immunology, Moscow, Russia.; The University of Hong Kong; Department of Paediatrics and Adolescent Medicine, University of Hong Kong, Hong Kong, China.; Division of Geriatric Medicine, Tours University Medical Center, Tours, France.; INSERM U1259, MAVIVH, Université de Tours, Tours, France.; Department of Neurology, Amsterdam Neuroscience, Amsterdam, Netherlands.; Department of Microbiology, Universidad de La Sabana, Chía, Colombia.; Service de Biologie Médicale, CHI Robert Ballanger, Aulnay-sous-Bois, France.; Hospices Civils de Lyon; Immunology department; Rockefeller University; Service de Médecine Intensive Réanimation, Hôpitaux Universitaires Henri Mondor, Assistance Publique-Hôpitaux de Paris (AP-HP), Paris, France.; Department of Nephrology and Transplantation, Necker University Hospital, APHP, Paris, France. 58INEM, INSERM U1151–CNRS UMR 8253, Paris University, Paris, France.; Genetics Institute, Tel Aviv Sourasky Medical Center, Tel Aviv University, Tel Aviv, Israel.; Sackler Faculty of Medicine, Tel-Aviv University, Tel-Aviv; Medical Intensive Care Unit, UZ Gasthuisberg & Laboratory for Clinical Infectious and Inflammatory Disorders, Depart-ment of Microbiology, Immunology and Transplantation, KU Leuven; University Hospitals Leuven; Department of Age-Related Healthcare, Tallaght University Hospital, Dublin, Ireland.; Department of Age-Related Healthcare, Tallaght University Hospital, Dublin, Ireland.; Department of Medical Gerontology, School of Medicine, Trinity College Dublin, Dublin, Ireland.; SIMR; Sharjah Institute for Medical Research, College of Medicine, University of Sharjah, Sharjah, United Arab Emirates.; Sorbonne Universités, UPMC Univ Paris 06, INSERM, CNRS, Centre d’Immunologie et des Maladies Infectieuses (CIMIParis UMRS 1135); Cerba HealthCare, Issy-les-Moulineaux, France.; Cerba HealthCare, Issy-les-Moulineaux, France.; Department of Pediatrics, King Fahad Hospital of the University, Al Khobar, Saudi Arabia.; Department of Clinical Laboratory, Hospital Universitari de Bellvitge, IDIBELL, Barcelona, Spain.; Department of Immunology, Hospital Universitari de Bellvitge, IDIBELL, Barcelona, Spain.; Department of Medicine, Division of Infectious Diseases, McGill University Health Centre, Montréal, QC, Canada.; The Research Institute of the McGill University Health Centre; Aarhus University Hospital; Institute of Biomedical Sciences, University of São Paulo; Faculdades Pequeno Príncipe, Instituto de Pesquisa Pelé Pequeno Príncipe, Curitiba, Brazil.; Shupyk National Healthcare University of Ukraine, Kyiv, Ukraine.; St. Giles Laboratory of Human Genetics of Infectious Diseases, Rockefeller Branch, Rockefeller University, New York, NY, USA.; Service de Médecine Interne, Hôpital universitaire Jean-Verdier AP-HP, Bondy, France.; École Polytechnique Fédérale de Lausanne; Hôpital Necker-Enfants Malades; Yale University School of Medicine; Laboratory of Human Genetics and Genomics, The Rockefeller University; Yale University School of Medicine; Diabetes Center, University of California San Francisco, San Francisco, CA, USA.; Rockefeller University; INSERM U1163; INSERM U1163; Biomedical Research Foundation, Academy of Athens; Institut National de la Santé et de la Recherche Médicale, Unité Mixte de Recherche (UMR) 1163; ICREA/ IDIBELL; Molecular Pathology Research Group, Institute of Biomedicine and Translational Medicine, University of Tartu; Aarhus University; Institute for Systems Biology, Seattle, WA, USA.; ADMA Biologics Inc., Ramsey, NJ, USA; University of Bordeaux, Inserm, Bordeaux Population Health Research Center, UMR 1219; Aix-Marseille University; INSERM CIC 1425, Paris, France.; APHP- Hôpital Bichat – Médecine Intensive et Réanimation des Maladies; Université de Paris, Université Paris-Saclay, UVSQ, INSERM UMS11, Villejuif, France.; Vall d’Hebron University Hospital; Hospital Universitari Vall d’Hebron (HUVH); APHP Sorbonne universite; Department of Internal Medicine, Hospital Universitari de Bellvitge, IDIBELL, Barcelona, Spain.; Université de Lille, INSERM, CHU de Lille, Institut Pasteur de Lille, U1011-EGID, F-59000 Lille, France.; IrsiCaixa; Aix Marseille Université; IHU Méditerranée Infection-MEPHI; Service de Biologie Clinique and UMR-S 1176, Hôpital Foch, Suresnes, France.; Feinstein Institutes for Medical Research, Northwell Health, Manhasset, NY, USA.; Department of Immunology, University Hospital of Gran Canaria Dr. Negrin, Canarian Health System, Las Palmas de Gran Canaria, Spain.; IRCCS Ospedale San Raffaele, San Raffaele Diabetes Research Institute, Via Olgettina 60, 20132 Milan; NIAID/NIH; NIAID, NIH; University of Tartu; Hiroshima University Graduate School of Biomedical and Health Sciences; INSERM; Rockefeller University; Rockefeller University; EFS; St. Giles Laboratory of Human Genetics of Infectious Diseases, Rockefeller Branch, Rockefeller University, New York, NY, USA.; Necker Medical School; Necker Medical School; INSERM

## Abstract

SARS-CoV-2 infection fatality rate (IFR) doubles with every five years of age from childhood onward. Circulating autoantibodies neutralizing IFN-α, IFN-ω, and/or IFN-β are found in ~20% of deceased patients across age groups. In the general population, they are found in ~1 % of individuals aged 20–70 years and in >4% of those >70 years old. With a sample of 1,261 deceased patients and 34,159 uninfected individuals, we estimated both IFR and relative risk of death (RRD) across age groups for individuals carrying autoantibodies neutralizing type I IFNs, relative to non-carriers. For autoantibodies neutralizing IFN-α2 or IFN-ω, the RRD was 17.0[95% CI:11.7–24.7] for individuals under 70 years old and 5.8[4.5–7.4] for individuals aged 70 and over, whereas, for autoantibodies neutralizing both molecules, the RRD was 188.3[44.8–774.4] and 7.2[5.0–10.3], respectively. IFRs increased with age, from 0.17%[0.12–0.31] for individuals <40 years old to 26.7%[20.3–35.2] for those ≥80 years old for autoantibodies neutralizing IFN-α2 or IFN-ω, and from 0.84%[0.31–8.28] to 40.5%[27.82–61.20] for the same two age groups, for autoantibodies neutralizing both molecules. Autoantibodies against type I IFNs increase IFRs, and are associated with high RRDs, particularly those neutralizing both IFN-α2 and -ω. Remarkably, IFR increases with age, whereas RRD decreases with age. Autoimmunity to type I IFNs appears to be second only to age among common predictors of COVID-19 death.

## Introduction

There have already been more than 250 million SARS-CoV-2 infections and at least five million deaths from COVID-19 worldwide. Interindividual clinical variability in the course of infection with SARS-CoV-2 is immense, ranging from silent infection in about 40% of cases to acute respiratory distress syndrome in ~3% of cases^1–3^. Death occurs in ~1 % of cases^4^. Age is the strongest epidemiological predictor of COVID-19 death, with the risk of death doubling every five years of age from childhood onward^4,5^. Men are also at greater risk of death than women^3,6^. The COVID Human Genetic Effort^7^ has shown that type I interferon (IFN) immunity is essential for protective immunity to respiratory infection with SARS-CoV-2^8–11^. We have reported that inborn errors of Toll-like receptor 3 (TLR3)-dependent type I IFN immunity can underlie life-threatening COVID-19 pneumonia in a small subset of patients^11^. Biochemically deleterious mutations of eight genes were found in 23 patients with critical COVID-19 (3.5% of 659 patients), including 18 patients under 60 years old. Remarkably, four patients, aged 25 to 50 years, had autosomal recessive (AR) deficiency of IFNAR1 or IRF7. Two other patients with AR IFNAR1 or TBK1 deficiency were independently reported^12,13^. The penetrance of those defects is unknown, but it is probably higher for AR than for autosomal dominant disorders. We then reported that X-linked recessive TLR7 deficiency accounted for 1.8% of cases of life-threatening COVID-19 in men under 60 years old^10,14^. The penetrance of this disorder is apparently high but incomplete, especially in children. Deficiencies of IFNAR1 and IRF7 blunt type I IFN immunity across cell types, whereas defects of the TLR3 and TLR7 pathway preferentially affect respiratory epithelial cells and plasmacytoid dendritic cells, respectively^10,15^.

We have also reported the presence of autoantibodies (auto-Abs) neutralizing high concentrations (10 ng/mL, with plasma diluted 1/10) of IFN-α2 and/or IFN-ω in about 10% of patients with critical COVID-19 pneumonia but not in individuals with asymptomatic or mild infection^9^. This finding has already been replicated in 13 other cohorts^16–29^. We then detected auto-Abs neutralizing lower, more physiological concentrations (100 pg/mL, with plasma diluted 1/10) of IFN-α2 and/or IFN-ω in 13.6% of patients with life-threatening COVID-19, and 18% of deceased patients^8^. The proportion of male patients was greater in patients with auto-Abs than in patients without auto-Abs^8,9^. In addition, 1.3% of patients with critical COVID-19 had auto-Abs neutralizing IFN-β (10 ng/mL, with plasma diluted 1/10), most without auto-Abs neutralizing IFN-α2 or IFN-ω. The prevalence of auto-Abs neutralizing IFN-α2 and/or IFN-ω in the general population increased with age, from 0.18% for 10 ng/mL and 1 % for 100 pg/mL in individuals between 18 and 69 years old to 3.4% for 10 ng/mL and 6.3% for 100 pg/mL for individuals over 80 years old^8^. The prevalence of auto-Abs against IFN-β did not increase with age. The crude odds ratios (ORs) for critical COVID-19 as opposed to asymptomatic or mild infection in auto-Ab carriers relative to non-carriers ranged from 3 to 67, depending on the type I IFNs recognized and the concentrations neutralized^8^. At least 12 lines of evidence strongly suggest that auto-Abs against type I IFNs are strong determinants of COVID-19 death (Table 1). The specific impact of these auto-Abs on COVID-19 mortality according to age and sex remains unknown and is of major interest, as both the prevalence of these auto-Abs and the risk of death increase with age and are higher in men. Here, we estimated the relative risk of COVID-19 death (RRD) and the SARS-CoV-2 infection fatality rate (IFR) for type I IFN auto-Ab carriers relative to non-carriers, by sex and age category.

## Methods

### Study design

We enrolled 1,261 patients aged 20 to 99 years old who died from COVID-19 pneumonia, and 34,159 controls from the adult general population from whom samples were collected before the COVID-19 pandemic, as previously described^8^. All subjects were recruited according to protocols approved by local institutional review boards (IRBs). Auto-Ab determinations were performed as described by Bastard *et al*.^8,46^, and were classified as neutralizing high concentrations (10 ng/ml) of IFN-α2, -ω, or –β, or low concentrations (100 pg/ml) of IFN-α2, or –ω (Additional Methods).

### RRDs and IFRs for carriers of neutralizing autoantibodies

We estimated the RRD in individuals carrying auto-Abs neutralizing type I IFNs relative to non-carriers, using large samples of patients who died from COVID-19 and of individuals from the general population. For each combination of auto-Abs, a Firth’s bias-corrected logistic regression model, including auto-Ab status, sex and age was fitted (Supplementary Table 1). For assessments of the effect of age and sex on the RRD due to auto-Abs, we added auto-Abs*sex and auto-Abs*age interaction terms to the Firth’s logistic regression model (Additional Methods). We estimated the IFR for carriers of neutralizing auto-Abs infected with SARS-CoV-2 (IFR_AAB_) following Bayes’ theorem, and using the age-dependent prevalence of auto-Abs in deceased patients and in the general population together with the reported age-specific IFR^4^ as detailed in Additional Methods.

## Results

### Patients and controls

We estimated the RRD of individuals carrying auto-Abs neutralizing type I IFNs relative to non-carriers by Firth’s logistic regression, using large samples of 1,261 patients who died from COVID-19 and 34,159 individuals from the general population from whom samples were collected before the pandemic. In this study design, in which controls are sampled from the baseline population regardless of disease status, the ORs obtained by logistic regression approximate to the RR in the absence of the assumption of rare disease^47^ (see Additional Methods). For auto-Abs neutralizing low concentrations (100 pg/mL) of IFN-α2 and/or IFN-ω, we used 1,121 patients who died from COVID-19, and 10,778 individuals from the general population (Table 2).

Assessments of auto-Abs neutralizing high concentrations (10 ng/mL) of IFN-α2 and/or IFN-ω were available for 1,094 deceased patients, and 34,159 individuals from the general population (Table 2). We also had assessments of auto-Abs neutralizing 10 ng/mL IFN-β for a subsample of 636 deceased patients and 9,126 individuals from the general population (Table 2). RRD was estimated by means of Firth’s bias-corrected logistic regression, considering death as a binary outcome and adjusting for sex and age in six classes (20–39, 40–49, 50–59, 60–69, 70–79, ≥80 years). For assessment of the effect of age and sex on RRD, we added auto-Abs*age and auto-Abs*sex interaction terms to the logistic model (see Methods and Additional Methods).

### RRD for carriers of auto-Abs neutralizing low concentrations of type I IFNs

**Figure 1:**
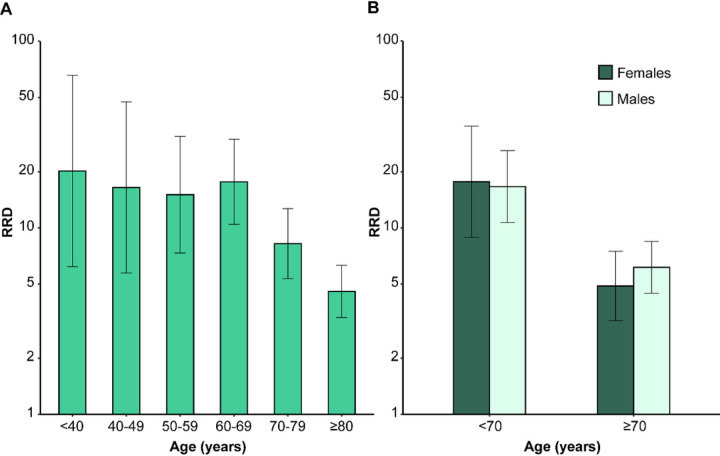
Relative risks of death associated with auto-Abs neutralizing low concentrations of IFN-α2 or -ω, by age and sex. RRDs for individuals with auto-Abs neutralizing low concentrations of IFN-α2 or IFN-ω relative to individuals without such auto-Abs, by age and sex. RRDs are displayed on a logarithmic scale (A) for six age classes, and (B) for male and female subjects under and over the age of 70 years. Vertical bars represent the 95% CI.

**Figure 2:**
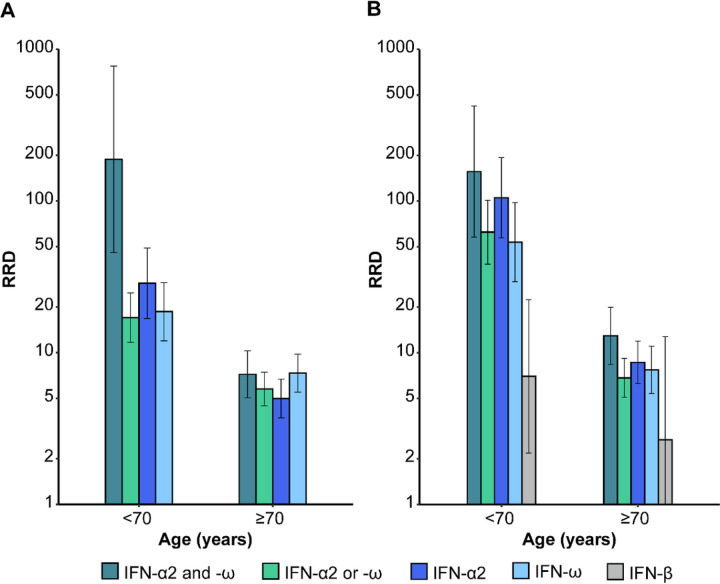
Relative risks of death associated with auto-Abs neutralizing various combinations of type I IFNs, by age. RRDs for individuals with auto-Abs neutralizing different combinations of type I IFNs relative to individuals without such auto-Abs, by age. RRDs are displayed on a logarithmic scale for individuals under and over 70 years of age with (A) auto-Abs neutralizing low concentrations of IFN-α2 and IFN-ω, IFN-α2 or IFN-ω, IFN-α2, IFN-ω, and (B) auto-Abs neutralizing high concentrations of IFN-α2 and IFN-ω, IFN-α2 or IFN-ω, IFN-α2, IFN-ω and IFN-β, relative to individuals without such combinations of auto-Abs. Vertical bars represent the 95% CI.

We first estimated the RRD for individuals carrying auto-Abs neutralizing low concentrations of IFN-α2 or IFN-ω. As expected, increasing age and maleness were highly significantly associated with greater risk of COVID-19 death (*P* values ≤10^−16^, Supplementary Table 1). Different age classes were used to test the interaction with the presence of auto-Abs, and the best fit was obtained with a two-age class model (20–69 and ≥70 years, Supplementary Table 2) with a significant effect of the auto-Abs*age interaction term (*P* value = 4×10^−6^). The RRD associated with auto-Abs did not vary significantly with sex (*P* value = 0.81). These interaction results are fully consistent with the distribution of RRD according to age (Fig. 1A) and sex (Fig. 1B), with a clear decrease in RRD after the age of 70 years, and no sex effect. Overall, the RRD for individuals carrying auto-Abs neutralizing IFN-α2 or IFN-ω decreased from 17.0 [95% CI: 11.7–24.7] before the age of 70 years to 5.8 [4.5–7.4] for individuals ≥70 years old (Fig. 2A, Supplementary Table 3). We then applied the same strategy to other combinations of auto-Abs neutralizing low concentrations of IFN, and observed similar age effects on RRDs (Supplementary Table 1). The presence of auto-Abs neutralizing both IFN-α2 and IFN-ω was associated with the highest RRD, estimated at 188.3 [45.8–774.4]) for individuals under the age of 70 years and 7.2 [5.0–10.3] for those over 70 years old (Fig. 2A, Supplementary Table 3).

### RRD for carriers of auto-Abs neutralizing high concentrations of type I IFNs

We then estimated the RRD for the presence *versus* the absence of auto-Abs neutralizing high concentrations (10 ng/mL) of type I IFN. The effect of age on RRD was similar to that observed with auto-Abs neutralizing low concentrations of type I IFN, with the use of two age classes providing the best fit (Supplementary Table 2 and 4). The RRDs associated with auto-Abs neutralizing high concentrations of type I IFNs were higher than those associated with auto-Abs neutralizing low concentrations, and also decreased with age (Fig. 2B, Supplementary Table 5). The RRD for carriers of IFN-α2 or IFN-ω auto-Abs decreased from 62.4 [38.4–101.3] before the age of 70 years to 6.8 [5.1–9.2] after the age of 70 years, whereas carriers of auto-Abs against both IFN-α2 and IFN-ω had the highest RRD, estimated at 156.5 [57.8–423.4] and 12.9 [8.4–19.9] for subjects <70 years and ≥70 years old, respectively (Fig. 2B, Supplementary Table 5). Interestingly, auto-Abs neutralizing high doses of IFN-β had the lowest RRD before 70 years (7.0 [2.2–22.4]), with no significant age-dependent association (*P* value = 0.37).

### IFR in individuals carrying auto-Abs neutralizing low concentrations of type I IFNs

**Figure 3:**
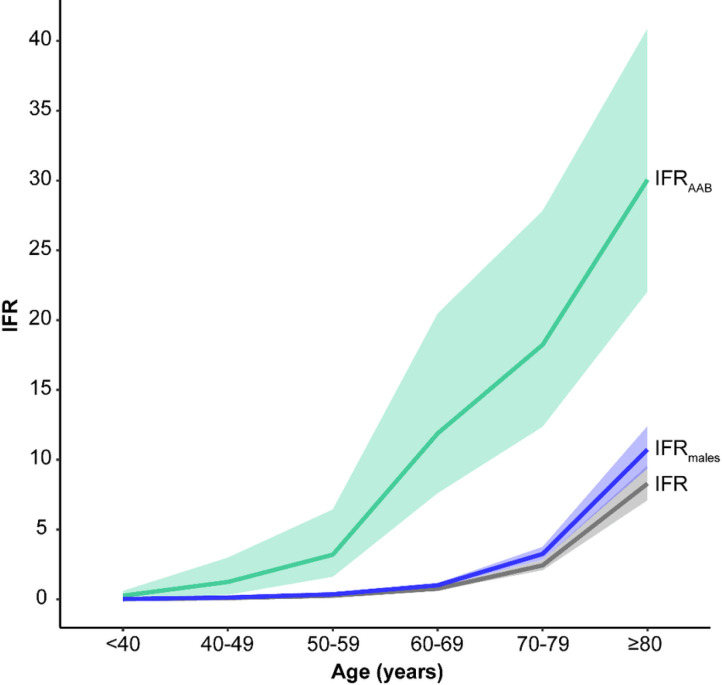
SARS-CoV-2 infection fatality rates by age. IFRs are provided in the general population for both sexes (gray) and for males only (blue) using the data of O’Driscoll et al. 4; IFR_AAB_ (green) are shown for individuals carriers of auto-Abs neutralizing low concentrations of IFN-α2 or IFN-ω. Auto-Abs against type I IFNs are associated with high RRDs and strongly increase IFR, to a much greater extent than maleness, and by inference than other classical common risk factors providing ORs of death similar to maleness (around 2) such as some comorbidities, or the most significant common genetic variant on chromosome 3^3^.

**Figure 4:**
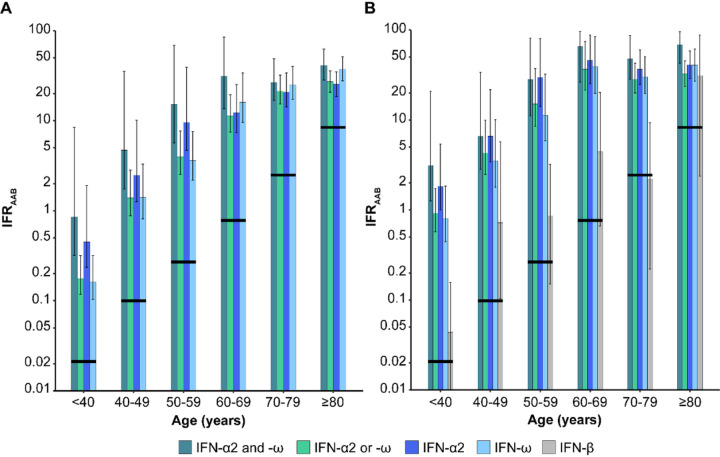
SARS-CoV-2 infection fatality rates for carriers of various combinations of neutralizing auto-Abs, by age. IFR_aab_ values (%) are displayed, on a logarithmic scale, by age, for individuals with (A) auto-Abs neutralizing low concentrations of IFN-α2 and IFN-ω, IFN-α2 or IFN-ω, IFN-α2, IFN-ω, and (B) auto-Abs neutralizing high concentrations of IFN-α2 and IFN-ω, IFN-α2 or IFN-ω, IFN-α2, IFN-ω and lFN-β. Vertical bars represent the 95% CI. Horizontal black lines represent the IFR provided by O’Driscoll *et al*^4^.

We then estimated the IFR in SARS-CoV-2-infected individuals carrying auto-Abs neutralizing low concentrations of type I IFNs. According to Bayes’ theorem, IFR_AAB_ can be expressed as a function of the age-dependent prevalence of auto-Abs in deceased patients and in the general population together with the reported age-specific IFR^4^ (see Supplementary Information). For all combinations of auto-Abs, the IFR_aab_ was much higher than the overall IFR. Figure 3 illustrates this much higher IFR for carriers of auto-Abs neutralizing low concentrations of IFN-α2 or IFN-ω; it exceeded 1 % and 10% for subjects over the ages of 40 and 60 years, respectively. Considering other combinations of auto-Abs, the highest IFR_AAB_ was observed for carriers of auto-Abs neutralizing both IFN-α2 and -ω, reaching 40.5% [27.8–61.2] in individuals over 80 years old (Fig. 4A and Supplementary Table 6). IFR_AAB_ values were similar for all other combinations of auto-Abs. For example, the IFR_AAB_ for individuals carrying auto-Abs neutralizing either IFN-α2 or -ω ranged from 0.17% [0.12–0.31] in individuals under 40 years old to 26.7% [20.3–35.2] in individuals over 80 years old. An exception was noted for the IFR_AAB_ of carriers of anti-IFN-α2 auto-Abs, which was 1.8 to 2.6 times higher than that for carriers of auto-Abs neutralizing IFN-α2 or -ω in subjects under 60 years old.. The IFR_AAB_ was also generally higher in male subjects than in female subjects, particularly in individuals carrying auto-Abs neutralizing both IFN-α2 and -ω (~2.7 times higher) (Supplementary Fig. 1).

### IFR in individuals carrying auto-Abs neutralizing high concentrations of type I IFNs

The age-, sex- and type I IFN-dependent patterns of IFR_AAB_ observed for carriers of auto-Abs neutralizing high concentrations of IFN-α2 and/or -ω were similar to those previously obtained for carriers of auto-Abs neutralizing low concentrations of these molecules, but with higher values. For example, IFR_AAB_ ranged from 3.1% [1.3–20.8] before 40 years of age to 68.7% [42.5–95.8] in those over 80 years old for carriers of auto-Abs neutralizing high concentrations of both IFN-α2 and -ω (Fig. 4B, Supplementary Table 7). IFR_AAB_ values were ~5 times higher in male than in female subjects, across all age groups and auto-Abs combinations (Fig. 2). For carriers of auto-Abs neutralizing IFN-β (tested only at high concentration), IFR_aab_ was lower (by a factor of six to 71) than for individuals under the age of 80 years with auto-Abs neutralizing IFN-α2 and/or -ω. It ranged from 0.04% [0.01–0.16] for individuals under the age of 40 years to 2.2% [0.2–9.3] for the 70–79 years age group. In the oldest age-class, IFR_AAB_ was 31.0% [2.4–88.1], similar to that for carriers of auto-Abs against IFN-α2 or -ω, albeit with a large confidence interval.

## Discussion

In this study, we estimated RR from the general population^47^ to obtain the RRDs associated with auto-Abs. We also used IFR values previously reported for the general population^4^ to estimate IFR_AAB_. We report high RRDs for carriers of auto-Abs neutralizing type I IFNs, ranging from 2.6 for auto-Abs neutralizing IFN-β (high concentration) in subjects over 70 years old to >150 for auto-Abs neutralizing both IFN-α2 and IFN-ω in subjects under 70 years old. For all types of auto-Abs, RRDs were three to 26 times higher in subjects under 70 years old than in older individuals. This is consistent with the increasing prevalence of auto-Abs in the general population with age (~1% under 70 years of age and >4% over 70 years of age), whereas the proportion of deceased patients with these auto-Abs is stable across age categories (~15–20%). The lower RRD observed in the elderly may be partly explained epidemiologically, by the larger contribution of other mortality risk factors, such as comorbid conditions, which become more frequent with increasing age. At the cellular level, aging is associated with immunosenescence, which may contribute to a defective innate and adaptive response to SARS-CoV-2 infection, thereby conferring a predisposition to severe COVID-19^48^. At the molecular level, global type I IFN immunity in the blood (plasmacytoid dendritic cells) and respiratory tract (respiratory epithelial cells) has been shown to decline with age^49–52^. These epidemiological, cellular, and molecular factors probably overlap. Thus, despite their increasing prevalence with age, auto-Abs against type I IFNs make a decreasing contribution to the risk of COVID-19 death with age due to the progressive development of additional age-dependent risk factors, including other mechanisms of type I IFN deficiency. However, for the very same reasons, IFR_AAB_ increases dramatically with age in patients with auto-Abs, reaching 68.7% for carriers of auto-Abs neutralizing high concentrations of both IFN-α2 and -ω.

RRD and IFR_AAB_ varied considerably with the IFNs recognized and the concentrations neutralized by auto-Abs. For most combinations involving auto-Abs against IFN-α2 and/or –ω, the neutralization of low concentrations was associated with a lower RRD and a lower IFR_AAB_ than the neutralization of high concentrations, suggesting that residual type I IFN activity may be beneficial in at least some patients. Blood IFN-α concentrations during acute asymptomatic or paucisymptomatic SARS-CoV-2 infection typically range from 1 to 100 pg/mL^8^. In addition, the presence of auto-Abs neutralizing both IFN-α2 and IFN-ω was associated with the highest RRD and IFR_AAB_ values. Interestingly, IFN-α2 and IFN-ω are encoded by two genes, *IFNA2* and *IFNW1,* that have been shown to have evolved under strong selective constraints^53^, consistent with their neutralization being harmful to the host. In addition, patients with auto-Abs against IFN-α2 have been shown to neutralize all 13 IFN-α subtypes^8,9^, rendering any potential IFN-α redundancy inoperative^8,9^. Accordingly, the IFR_AAB_ values for carriers of auto-Abs against IFN-α2 were higher than those for carriers of auto-Abs against IFN-ω in subjects under 60 years of age. In older age groups, this difference tended to disappear, consistent with the lower impact of auto-Abs in the elderly, as discussed above. Finally, auto-Abs neutralizing IFN-β were less common, and associated with lower RRD and IFR_AAB_ values (by about one order of magnitude) than auto-Abs against IFN-α2 and/or IFN-ω, in all age groups except the over-80s. This less deleterious effect of auto-Abs neutralizing IFN-β is consistent with a mouse study showing that the blockade of IFN-β alone does not alter the early dissemination of lymphocytic choriomeningitis virus^54^. Overall, auto-Abs against type I IFNs are associated with very high RRD and IFR values, and the magnitude of this effect is much larger than that of other known common risk factors apart from age, such as maleness (Fig. 4), comorbidities, or the most significant common genetic variant on chromosome 3, all of which have been associated to life-threatening COVID-19 with ORs of about 2^3^.

Despite the lower prevalence of these auto-Abs in younger than in older individuals, the much higher IFR_aab_ observed in individuals with these auto-Abs suggests that the testing of infected individuals in all age groups is warranted. Particular attention should be paid to patients, especially children, with known autoimmune or genetic conditions associated with the production of auto-Abs against type I IFNs. Early treatments could be provided^55^, including monoclonal antibodies^56^, new antiviral drugs, and/or IFN-β in the absence of auto-Abs against IFN-β^57,58^. Rescue treatment by plasma exchange is a therapeutic option in patients who already have pneumonia^30^. A screening of uninfected elderly people could be considered, given that these auto-Abs are found in 4% of individuals over 70 years old. Carriers of auto-Abs should be vaccinated against SARS-CoV-2 as a priority, and should benefit from a booster, whatever their age, and ideally from a monitoring of their antibody response to the vaccine. They should not receive live-attenuated vaccines, including the yellow fever vaccine (YFV-17D) and anti-SARS-CoV-2 vaccines based on the YFV-17D backbone^46^. In cases of SARS-CoV-2 infection, vaccinated patients should be closely monitored. As SARS-CoV-2 vaccination coverage increases and mortality due to COVID-19 decreases over time, it will be important to re-evaluate the risk of fatal COVID-19 in vaccinated individuals with and without auto-Abs. It is currently unclear whether these auto-Abs impair antibody responses to vaccines, and whether a vaccine-triggered antibody response can overcome type I IFN deficiency in response to large or even medium-sized viral inocula. Finally, further investigations are required to determine the contribution of these auto-Abs to other severe viral diseases, and to elucidate the mechanisms underlying their development, which may be age-dependent. In the meantime, auto-Abs against type I IFNs should be considered as a leading common predictor of life-threatening COVID-19 after age, as their detection has a much greater predictive value for death, and, by inference, hospitalization and critical COVID-19, than sex, comorbidities, and common genetic variants (Fig. 3).

## Supplementary Material

Supplement 1

## Figures and Tables

**Table 1.** Lines of evidence suggesting that auto-Abs against type I IFNs are strong determinants of the risk of life-threatening COVID-19.

Evidence	Examples	References
Auto-Abs against type I IFNs are present before SARS-CoV-2 infection	In patients for whom a sample collected before the COVID-19 pandemic was available, the auto-Abs were found to pre-exist infection	^30^
	These auto-Abs are found in the uninfected general population, and their prevalence increases after the age of 65 years	^8^
Auto-Abs are associated with COVID-19 severity	Patients with inborn errors underlying these auto-Abs from infancy onward (e.g. APS-1) have a very high risk of developing critical COVID-19 pneumonia	^30^
	The population of patients with critical disease includes a higher proportion of individuals producing these auto-Abs than the population of patients with silent or mild infection (ORs depending on the nature, number, and concentrations of type I IFN neutralized)	^8^
	The results concerning the proportions of critical cases with auto-Abs against type I IFNs have already been replicated in >15 different cities (Americas, Europe, Asia)	^16–29^
Auto-Abs against type I IFNs neutralize host antiviral activity	These auto-Abs neutralize the antiviral activity of type I IFNs against SARS-CoV-2 *in vivo*	^9^
	These auto-Abs are found *in vivo* in the blood of SARS-CoV-2-infected patients, where they neutralize type I IFN	^31^
	These auto-Abs are found *in vivo* in the respiratory tract of patients, where they neutralize type I IFN	^32–34^
	A key virulence factor of SARS-CoV-2 *in vitro* is its capacity to impair type I IFN immunity	^35^
	Animals with type I IFN deficiency develop critical disease, including animals treated with mAbs that neutralize type I IFNs	^36^
Auto-Abs against cytokines are clinical phenocopies of the corresponding inborn errors	Patients with auto-Abs against type I IFNs are phenocopies of IFNAR1^−/−^, IFNAR2^−/−^, and IRF7^−/−^ patients with critical COVID-19 pneumonia	^11^
	Patients with auto-Abs against IL-6, IL-17, GM-CSF, and type II IFN are phenocopies of the corresponding inborn errors and underlie staphylococcal disease, ucocutaneous candidiasis, nocardiosis, and mycobacterial diseases, respectively	^37–45^

**Table 2.** Characteristics of the general population cohort and of the cohort of patients who died from COVID-19, by age, sex and autoantibody status

	Neutralization 100 pg/mL		Neutralization 10 ng/mL	
	
Characteristics	General Population (*N*=10,778)	Deceased Patients (*N*=1,121)	General Population (*N*=34,159)	Deceased Patients (*N*=1,094)

**Male - no. (%)**	5,429 (50.4)^a^	821 (73.2)	17,859 (52.3)	805 (73.5)
**Mean age**^a^ **±SD - yr**	62.3 ±17.2	70.7 ±13.0	52.7 ±18.2	70.6 ±13.1
**Age distribution - no. (%)**				
20–39 yr	1,251 (11.6)	17 (1.5)	9,102 (26.6)	15 (1.4)
40–49 yr	1,459 (13.5)	43 (3.8)	5,403 (15.8)	47 (4.3)
50–59 yr	1,736 (16.1)	144 (12.8)	6,414 (18.9)	152 (13.9)
60–69 yr	2,475 (23.0)	307 (27.4)	6,881 (20.1)	289 (26.4)
70–79 yr	1,790 (16.6)	307 (27.4)	3,721 (10.9)	296 (27.1)
≥80 yr	2,067 (19.2)	303 (27.0)	2,638 (7.7)	295 (27.0)
**Auto-Ab - no. of carriers (%)**				
IFN-α2 and IFN-ω	65 (0.6)	102 (9.1)	45 (0.1)	75 (6.8)
IFN-α2 or IFN-ω	246 (2.3)	203 (18.1)	181 (0.5)	130 (11.9)
IFN-α2	151 (1.4)	140 (12.5)	117 (0.3)	118 (10.8)
IFN-ω	160 (1.5)	165 (14.7)	109 (0.3)	87 (8.0)
IFN-β^b^	NA	NA	24 (0.3)	6 (0.9)

SD standard deviation.

NA not available.

a Age is given in years and corresponds to age at the time of recruitment for members of the general population cohort (controls) and age at death for COVID-19 patients.

b IFN-β neutralization experiments were performed only for a concentration of 10 ng/mL, on 9,126 individuals (49.5% male, mean age 60.6 years) from the general population and 636 COVID-19 patients (71.1% male, mean age 72.9 years).
